# Systematic Review on Abdominal Penetrating Atherosclerotic Aortic Ulcers: Outcomes of Endovascular Repair

**DOI:** 10.1177/15266028231157636

**Published:** 2023-03-04

**Authors:** Johannes Hatzl, Dittmar Böckler, Jonathan Fiering, Samuel Zimmermann, Moritz Sebastian Biscshoff, Eva Kalkum, Rosa Klotz, Christian Uhl

**Affiliations:** 1 Department of Vascular and Endovascular Surgery, University Hospital Heidelberg, Heidelberg, Germany; 2 Institute of Medical Biometry and Informatics, University of Heidelberg, Heidelberg, Germany; 3 Study Center of the German Society of Surgery (SDGC), University of Heidelberg, Heidelberg, Germany

**Keywords:** covered stents, endovascular repair, ulcer, penetrating aortic ulcer, EVAR

## Abstract

**Purpose:**

To systematically review existing evidence on outcomes of endovascular repair of abdominal atherosclerotic penetrating aortic ulcers (PAUs).

**Material and Methods:**

Cochrane Central Registry of Registered Trials (CENTRAL), MEDLINE (via PubMed), and Web of Science databases were systematically searched. The systematic review was performed according to the Preferred Reporting Items for Systematic Reviews and Meta-Analysis protocol (PRISMA-P 2020). The protocol was registered in the international registry of systematic reviews (PROSPERO CRD42022313404). Studies reporting on technical and clinical outcomes of endovascular PAU repair in 3 or more patients were included. Random effects modeling was used to estimate pooled technical success, survival, reinterventions, and type 1 and type 3 endoleaks. Statistical heterogeneity was assessed using the I^2^ statistic. Pooled results are reported with 95% confidence intervals (CIs). Study quality was assessed using an adapted version of the Modified Coleman Methodology Score.

**Results:**

Sixteen studies including 165 patients with a mean/median age ranging from 64 to 78 years receiving endovascular therapy for PAU between 1997 and 2020 were identified. Pooled technical success was 99.0% (CI: 96.0%-100%). In all, 30-day mortality was 1.0% (CI: 0%-6.0%) with an in-hospital mortality of 1.0% (CI: 0.0%-13.0%). There were no reinterventions, type 1, or type 3 endoleaks at 30 days. Median/mean follow-up ranged from 1 to 33 months. Overall, there were 16 deaths (9.7%), 5 reinterventions (3.3%), 3 type 1 (1.8%), and 1 type 3 endoleak (0.6%) during follow-up. The quality of studies was rated low according to the Modified Coleman score at 43.4 (+/- 8.5) of 85 points.

**Conclusion:**

There is low-level evidence on outcomes of endovascular PAU repair. While in the short-term endovascular repair of abdominal PAU seems safe and effective, mid-term and long-term data are lacking. Recommendations with regard to treatment indications and techniques in asymptomatic PAU should be made cautiously.

**Clinical Impact:**

This systematic review demonstrated that evidence on outcomes of endovascular abdominal PAU repair is limited. While in the short-term endovascular repair of abdominal PAU seems safe and effective, mid-term and long-term data are lacking. In the context of a benign prognosis of asymptomatic PAU and lacking standardization in current reporting, recommendations with regard to treatment indications and techniques in asymptomatic PAUs should be made cautiously.

## Introduction

The term penetrating aortic ulcer (PAU) was defined in 1986 by Stanson et al.^1^ based on histopathological examination as an atherosclerotic lesion with ulceration that penetrates the internal elastic lamina and allows hematoma formation within the media of the aortic wall.^2^ More recently, the term PAU has been widely used to label ulcer-like lesions of the aorta in the presence of atherosclerosis. The European Society for Vascular Surgery (ESVS) 2019 Clinical Practice Guidelines on the Management of Abdominal Aorto-iliac Artery Aneurysms recommend serial imaging surveillance in uncomplicated cases and repair in patients with so called complicated abdominal PAU.^3^ The term “complicated PAU” thereby refers to a co-existing extra-aortic hematoma, embolization symptoms, recurrent pain, a PAU that initially measures >20 mm in width (length of the intimal defect at the ulcer site), or >10 mm in depth or progression of total abdominal aortic diameter.^3^ Recent evidence suggests a benign course of asymptomatic PAU with a low risk of disease progression, which re-raises the question of treatment indication and associated risks in these patients.^4^ Even though endovascular repair has been established for more than 2 decades, studies reporting outcomes of endovascular repair for abdominal PAU are scarce and the last review focusing on endovascular repair of abdominal PAU was published almost 10 years ago in 2013.^5^ Since then, additional literature has been published. This article systematically reviews existing evidence on outcomes of endovascular repair of abdominal atherosclerotic aortic PAUs.

## Materials and Methods

### Search Strategy

The systematic review was performed according to the Preferred Reporting Items for Systematic Reviews and Meta-Analysis protocol (PRISMA-P 2020).^6^ The protocol was registered in the international prospective registry of systematic reviews (PROSPERO CRD42022313404).

A systematic literature search was conducted to identify all relevant studies reporting outcomes of endovascular repair for abdominal PAU. Cochrane Central Registry of Registered Trials (CENTRAL), MEDLINE (via PubMed), and Web of Science databases were systematically searched. Medical Subject Headings (MeSH) terms as well as free text terms and Boolean operators were used. There were no language restrictions applied. A reference management software was used to conduct the analysis.^7^ The search strategy was developed with the assistance of a methodological expert in systematic search strategies (E.K.) according to the Population, Intervention, Comparison, Outcomes and Study (PICOS) criteria.^7^ The search was conducted on February 24, 2022.

### Study Selection

All potentially eligible records were reviewed independently by 2 authors (J.H., J.F.). The detailed search strategy is given in supplementary document 1. All types of studies were included. First, the titles and abstracts were screened. All relevant articles were retrieved, and full texts were independently reviewed. Studies that reported at least one of the relevant outcome criteria (technical success, survival, reinterventions, endoleaks) on 3 or more patients that were treated with endovascular repair for abdominal PAU were included. Mycotic PAUs were excluded. Any disagreements were resolved through discussion and consensus. If required, a third person (C.U.) was consulted. Duplicates were identified and excluded.

### Data Extraction

The 2 reviewers (J.H. and J.F.) independently performed data extraction using a standard data extraction form. If studies reported results of endovascular and open repair, data were extracted and analyzed for the endovascular subgroup only. The reviewers extracted: Authors, year of publication, study design, study period, sample size, mean and/or median age, sex, cardiovascular risk factors and comorbidity, clinical presentation, angio-morphological details, technical success rates of endovascular repair, device selection, mean, and/or median follow-up period as well as survival, reinterventions, and endoleaks each at 30 days, 1, and 3 years. Additionally, overall mortality, reintervention rates, and endoleak rates were extracted. Technical success was defined as successful delivery of the device and removal of the delivery system as well as freedom from endoleak types 1 and 3 as well as patency of the grafts at completion according to respective reporting standards.^8^ Reinterventions were defined as procedures aimed at maintaining aneurysm exclusion or distal perfusion. Endoleaks were defined according to established standards.^9^ Detailed analysis of adjunctive procedures could not be included due to heterogeneity in reporting. Outcome criteria selection for the purpose of this review was in accordance with pragmatic minimum reporting standards for endovascular aortic aneurysm repair.^10^

### Quality Assessment

Quality assessment was performed using the Modified Coleman Methodology Score which was adapted for the purposes of the present study and is attached in its adapted version in the supplementary document 2. The score evaluates various sources of bias and common shortcomings of observational studies in surgery. The score has been predominantly used in systematic reviews in orthopedic surgery and more recently in endovascular surgery as well.^11,12^ From the original version, the assessment of postoperative rehabilitation as well as procedures of assessing outcomes were excluded to accommodate to the present review and endovascular surgery, which left the following categories for analysis: Part A: (1) study size, (2) mean/median follow-up, (3) surgical approach, (4) type of study, (5) description of diagnosis, (6) description of surgical technique, and Part B: (7) outcome criteria, (8) description of study selection process. Scoring of all included studies was performed independently by 2 reviewers (J.H. and J.F.). A mean of the scores of both observers for every category was calculated and added up to a total score. The maximum score of the modified version was 85 points with a potential minimum score of 0.

### Statistical Analysis

Random effects modeling was used to estimate the technical success rate as well as survival and the incidence of reinterventions and type 1 and type 3 endoleaks at 1 month. Statistical heterogeneity was assessed using the I^2^ statistic. Pooled results are reported with 95% confidence intervals. Survival at 12, and 36 months as well as reinterventions and endoleaks beyond 1 month are reported descriptively due to the paucity and heterogeneity of available data. Statistical analyses were performed using R^13^ in RStudio,^14^ employing the package meta.^15^ Summary tables of random effect models and forest plots should be regarded as a supplementary visualization and overview for a descriptive interpretation of combined results.

## Results

**Figure 1.**
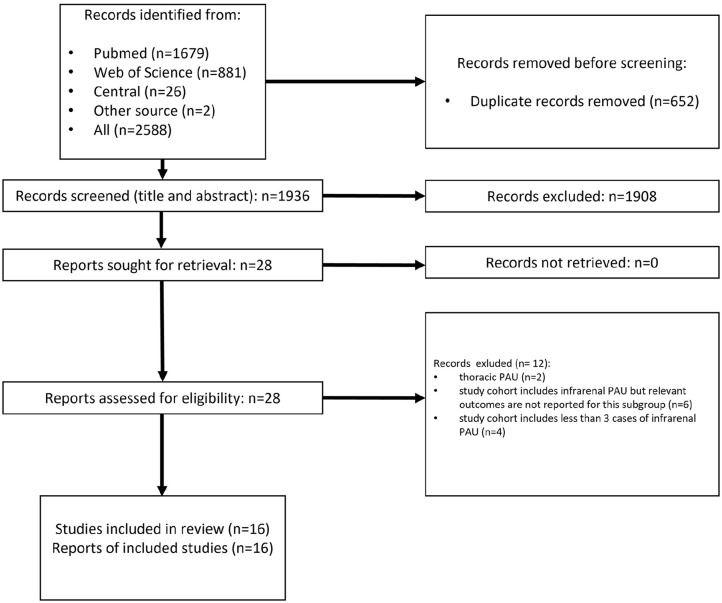
Flowchart of the study selection process according to the Preferred Reporting Items for Systematic Reviews and Meta-Analysis protocol (PRISMA-P 2020).^6^

Applying the search strategy resulted in 2588 hits. Following deletion of duplicates there were 1936 results. Screening of titles and abstracts left 28 results for full-text assessment. After full-text review, 16 studies reporting at least one relevant outcome criterion of endovascular management of abdominal aortic PAU, including 3 or more patients, were identified. There were 4 studies that were identified by 1 author but not the other and vice versa. After thorough discussion involving also the third author (C.U.), a consensus was reached that these studies fulfilled the inclusion criteria. Twelve studies were excluded for the following reasons: endovascular repair of thoracic PAU (N=2), studies included a subgroup of abdominal PAU but did not report relevant outcomes for this subgroup (N=6), and studies including less than 3 patients with abdominal PAU (N=4). A flow chart representing study selection is presented in Figure 1. Included studies are presented in Table 1.

**Table 1.** Citations, Study Designs, Number of Centers, Time Periods, and Sample Sizes of Included Studies.

ID	Citations	Study design	Centers (N)	Time period	Sample size (N)
1	Batt et al.^16^	Retrospective	1	2000-2003	3
2	Georgiadis et al.^17^	Prospective	2	2004-2012	19
3	Hyhlik-Dürr et al.^18^	Retrospective	1	1997-2009	20
4	Jones et al.^19^	Retrospective	1	2004-2011	5
5	Kazan et al.^20^	Retrospective	1	2011	3
6	Kruszyna et al.^21^	Retrospective	1	2017-2019	13
7	Palombo et al.^22^	Retrospective	1	2005-2009	3
8	Piffaretti et al.^23^	Retrospective	1	2002-2006	13
9	Stana et al.^24^	Retrospective	8	2017-2020	40
10	Tsuji et al.^25^	Retrospective	1	1999-2002	4
11	Wagenhäuser et al.^26^	Retrospective	4	2013-2020	18
12	Yao et al.^27^	Retrospective	1	2016-2018	4
13	Yoshida et al.^28^	Retrospective	2	2010-2020	4
14	Zhang et al.^29^	Retrospective	1	2018-2020	6
15	Sensi et al.^30^	Retrospective	1	2001-2005	6
16	Fyntanidou et al.^31^	Retrospective	1	2008	4
Overall				1997-2020	165

Overall, 16 studies including 165 patients with abdominal aortic PAU receiving endovascular therapy between 1997 and 2020 were identified. All but one study were retrospective observational studies. Four studies included patients from multiple sites, while the remaining 12 studies were single center studies.

### Quality Assessment

The quality of studies was rated according to the modified coalman score by 2 observers at 43.4 (±8.5) of 85 points, which is indicative of a low quality of evidence. Most points were lost due to small sample sizes, limited follow-up periods, and retrospective study designs. Details are presented in Table 2.

**Table 2.** Modified Coleman Methodology Score, Adapted for Endovascular Therapy of Penetrating Aortic Ulcers. The Applied Score Is Detailed in Supplementary Document 2.

	Batt et al.^16^	Georgiadis et al.^17^	Hyhlik-Dürr et al.^18^	Jones et al.^19^	Kazan et al.^20^	Kruszyna et al.^21^	Palombo et al.^22^	Piffaretti et al.^23^	Stana et al.^24^	Tsuji et al.^25^	Wagenhäuser et al.^26^	Yao et al.^27^	Yoshida et al.^28^	Zhang et al.^29^	Sensi et al.^30^	Fyntanidou et al.^31^	Mean (± SD)
Part A																	
Sample size *(max. 10)*	0	0	4	0	0	0	0	0	4	0	0	0	0	0	0	0	0.5 (1.3)
Follow-up *(max. 15)*	5	10	5	0	0	0	0	10	5	5	5	15	5	0	5	5	4.7 (4.1)
Surgical approach *(max 10)*	10	10	10	10	10	10	10	10	10	10	10	10	0	10	10	10	9.4 (2.4)
Type of study *(max. 15)*	0	15	0	0	0	0	0	0	0	0	0	0	0	0	0	0	0.9 (3.6)
Diagnosis *(max. 5)*	5	5	4	4	5	5	5	5	5	5	5	5	5	5	5	5	4.9 (0.3)
Surgical technique *(max 5)*	4	5	5	5	5	5	5	5	5	5	5	5	5	5	5	5	4.9 (0.2)
Total Score Part A *(max. 60)*	24	45	28	19	20	20	20	30	29	25	25	35	15	20	25	25	25.3 (7.0)
Part B																	
Outcome criteria *(max. 10)*	7.0	8.5	8.5	7.0	3.0	8.5	7.0	8.5	8.5	8.5	8.5	6.5	5.5	7.0	7.0	8.5	7.4 (1.5)
Selection process *(max. 15)*	11.5	11.5	11.5	9	6.5	11.5	11.5	11.5	9	11.5	11.5	11.5	9	11.5	11.5	11.5	10.7 (1.5)
Total Score Part B *(max. 25)*	18.5	20.0	20.0	16.0	9.5	20.0	18.5	20.0	17.5	20.0	20.0	18.0	14.5	18.5	18.5	20.0	18.1 (2.7)
Total Score Part A and Part B *(max. 85)*	42.5	65.0	48.0	35.0	29.5	40.0	38.5	50.0	46.5	45.0	45.0	53.0	29.5	38.5	43.5	45.0	43.4 (8.5)

Mean values of both observers’ scores (J.H., J.F.) are presented for each category. Total score for each category and total scores for Part A and Part B is presented as mean (±standard deviation).

### Demographics, Cardiovascular Risk Factors and Comorbidity

In 146 of 165 included patients (88%), data on sex could be extracted. Of these, 30 patients had female sex (30/146, 20.5%). Measures of central tendency for age (mean or median) ranged from 64 to 78 years. The most frequently reported cardiovascular risk factor was hypertension (96%) followed by hyperlipidemia (55%), smoking (49.0%), and diabetes mellitus (22%). Atherosclerotic co-manifestations were frequent with coronary artery disease (CAD, 37%), and peripheral arterial occlusive disease (PAOD, 35.0%) followed by cerebrovascular disease (CVD, 19%). Detailed information on demographics, cardiovascular risk factors, and comorbidities are presented in Table 2.

### Clinical Presentation

In 122 of 165 patients (74%), information is given about the clinical presentation of patients. Out of 122 patients, 55 (55/122, 45%) were reported to have presented with symptoms originating from abdominal PAU. The most common symptom was pain without signs of rupture (42/55, 76.4%). In all, 13 patients (13/55, 23.6%) presented with rupture of which 8 (8/55, 15%) were described to be associated with severe hypotension. Peripheral embolization was the least common symptom with only 4 cases (4/55, 7%). Clinical presentation is summarized in Table 3.

**Table 3.** Demographics, Cardiovascular Risk Factors, Comorbidity, and Clinical Presentation.

ID	Citations	Sample size (N)	Age		Male sex (N, %)	Smoking (N, %)	COPD* (N, %)	Hyperlipidemia (N, %)	CAD* (N, %)	CVD* (N, %)	PAOD* (N, %)	Hypertension (N, %)	Diabetes mellitus (N, %)	Renal insufficiency (N, %)	Symptoms (N, %)	Ruptures (N, %)	Peripheral embolization (N, %)	Pain (N, %)
1	Batt et al.^16^	3	72^a^	N	2	NR	NR	NR	NR	NR	NR	NR	NR	NR	2	0	1	1
				%	67	NR	NR	NR	NR	NR	NR	NR	NR	NR	67	0	33	33
2	Georgiadis et al.^17^	19	70^a^	N	18	13	1	11	11	3	9	18	3	NR	14	6	0	8
				%	95	68	5	58	58	16	47	95	16	NR	74	32	0	42
3	Hyhlik-Dürr et al.^18^	20	72^a^	N	17	13	1	NR	8	NR	NR	19	8	4	5	2	0	3
				%	85	65	5	NR	40	NR	NR	95	40	20	25	10	0	15
4	Jones et al.^19^	5	76^a^	N	4	1	NR	3	NR	NR	NR	3	NR	1	2	0	0	2
				%	80	20	NR	60	NR	NR	NR	60	NR	20	40	0	0	40
5	Kazan et al.^20^	3	76^a^	N	2	NR	NR	NR	NR	NR	NR	NR	NR	NR	3	0	0	3
				%	67	NR	NR	NR	NR	NR	NR	NR	NR	NR	100	0	0	100
6	Kruszyna et al.^21^	13	NR	N	NR	NR	NR	NR	NR	NR	NR	NR	NR	NR	NR	NR	NR	NR
				%	NR	NR	NR	NR	NR	NR	NR	NR	NR	NR	NR	NR	NR	NR
7	Palombo et al.^22^	3	64^a^	N	3	NR	NR	NR	NR	NR	NR	NR	NR	NR	0	0	0	0
				%	100	NR	NR	NR	NR	NR	NR	NR	NR	NR	0	0	0	0
8	Piffaretti et al.^23^	13	77^a^	N	12	NR	6	1	2	3	NR	13	5	2	5	1	3	1
				%	92	NR	46	8	15	23	NR	100	38	15	38	8	23	8
9	Stana et al.^24^	40	74^b^	N	25	NR	4	NR	12	NR	16	29	9	12	13	2	0	11
				%	63	NR	10	NR	30	NR	40	73	23	30	33	5	0	28
10	Tsuji et al.^25^	4	78^b^	N	4	NR	NR	NR	2	NR	NR	4	NR	NR	3	0	0	3
				%	100	NR	NR	NR	50	NR	NR	100	NR	NR	75	0	0	75
11	Wagenhäuser et al.^26^	18	76^b^	N	14	3	4	14	6	NR	2	16	0	5	NR	NR	NR	NR
				%	78	17	22	78	33	NR	11	89	0	28	NR	NR	NR	NR
12	Yao et al.^27^	4	NR	N	4	NR	NR	NR	NR	NR	NR	NR	NR	NR	4	0	0	4
				%	100	NR	NR	NR	NR	NR	NR	NR	NR	NR	100	0	0	100
13	Yoshida et al.^28^	4	65^b^	N	2	4	NR	3	1	NR	NR	3	1	0	4	0	0	4
				%	50	100	NR	75	25	NR	NR	75	25	0	100	0	0	100
14	Zhang et al.^29^	6	NR	N	NR	NR	NR	NR	NR	NR	NR	NR	NR	NR	NR	NR	NR	NR
				%	NR	NR	NR	NR	NR	NR	NR	NR	NR	NR	NR	NR	NR	NR
15	Sensi et al.^30^	6	78^a^	N	5	1	2	2	4	NR	NR	6	0	NR	NR	NR	NR	NR
				%	83	17	33	33	67	NR	NR	100	0	NR	NR	NR	NR	NR
16	Fyntanidou et al.^31^	4	67^b^	N	4	NR	1	3	1	NR	NR	4	NR	1	4	2	0	2
				%	100	NR	25	75	25	NR	NR	100	NR	25	100	50	0	50
Overall		165	64 - 78	N	116 of 146	35 of 72	19 of 120	38 of 69	47 of 128	6 of 32	27 of 77	115 of 120	26 of 120	25 of 104	55 of 122	13 of 122	4 of 122	42 of 122
				%	80	49	16	55	37	19	35	96	22	24	45	11	3	34

Data is presented as N (%).

Abbreviations: CAD, coronary artery disease; COPD, chronic-obstructive pulmonary disease; CVD, cerebrovascular disease; PAOD, peripheral arterial occlusive disease; NR, not reported.

Median^a^ or mean^b^.

### PAU Morphology

Maximum aortic diameter was reported for 49 of 165 included patients (29.7%) with measure of central tendency (mean or median) ranging from 21.3 mm to 44.0 mm, respectively. Penetrating aortic ulcer depth could be extracted for 65 patients (39.4%) and central measures of tendency ranged from 7.3 mm to 19.6 mm. PAU width was reported in only 1 study comprising 19 patients (11.5%) and measured a median of 9.5 mm. PAU morphology is presented in Table 4.

**Table 4.** Morphology of Penetrating Aortic Ulcers.

ID	Citation	N	Maximum aortic diameter	PAU width	PAU depth
1	Batt et al.^16^	3	NR	NR	NR
2	Georgiadis et al.^17^	19	44^a^ (IQR, 30-58)	9.5^a^ (IQR, 8-16)	NR
3	Hyhlik-Dürr et al.^18^	20	NR	NR	NR
4	Jones et al.^19^	5	NR	NR	NR
5	Kazan et al.^20^	3	NR	NR	NR
6	Kruszyna et al.^21^	13	NR	NR	15.5^a^ (range: 9-15)
7	Palombo et al.^22^	3	NR	NR	NR
8	Piffaretti et al.^23^	13	NR	NR	NR
9	Stana et al.^24^	40	NR	NR	19.6^b^ (±1.8)
10	Tsuji et al.^25^	4	NR	NR	NR
11	Wagenhäuser et al.^26^	18	36.1^b^(± 6.8)	NR	NR
12	Yao et al.^27^	4	NR	NR	NR
13	Yoshida et al.^28^	4	NR	NR	NR
14	Zhang et al.^29^	6	21.3^b^(±1.6)	NR	7.3^b^ (±2.8)
15	Sensi et al.^30^	6	23^a^ (range: 18-40)	NR	15.5^a^ (range: 10-20)
16	Fyntanidou et al.^31^	4	NR	NR	NR

Abbreviations: NR, Not reported; PAU, penetrating aortic ulcer.

Data are presented as median^a^ (with range or interquartile range (IQR) depending on availability of data) or mean^b^ (± standard deviation).

### Technical Results

**Figure 2.**
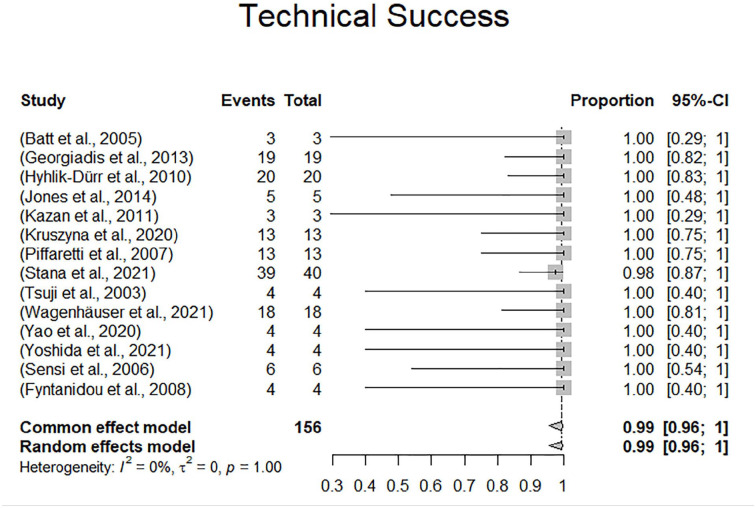
Forest plot displaying technical success.

Device selection was reported with varying degree of detail in 14 of 16 studies including 146 of 165 patients (88.5%). Endovascular aneurysm repair devices were used in 81 patients (81/146, 55.5%) of which 6 had an aorto-aortic tube configuration (6/81, 7.4%), 16 had aorto-uniiliac configuration (16/86, 19.8%), and the remaining 59 had aortobiiliac configuration (59/86, 72.8%). More recently, endovascular repair using covered stents (BeGraft stent graft system, Bentley InnoMed, Hechingen, Germany) was reported in 2 studies including 53 patients (53/146, 36.3%). One study reported a parallel stent graft technique in 4 patients (4/146, 2.7%). The remaining 8 patients (8/146, 5.5%) were treated using cuffs, iliac extensions, covered stents, or unspecified ancillary components. Technical success of endovascular repair was reported in 14 of 16 studies comprising 156 of 165 patients. All studies except for one exhibited 100% technical success rates, resulting in a pooled technical success rate according to the random effects model of 99.0% (96.0%-100%). The 1 case with technical failure was attributed to intraoperative aortic rupture after deployment of a BeGraft stent (Bentley InnoMed, Hechingen, Germany) with extensive oversizing in a severely calcified aorta. Devices and configurations are presented in supplementary Table 1. The forest plot for technical success is displayed in Figure 2.

### Clinical Results

#### Survival at 1 month and in-hospital mortality

**Figure 3.**
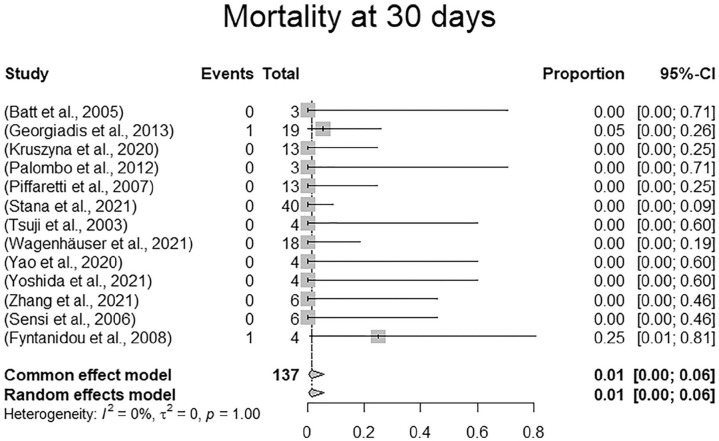
Forest plot displaying 30-day mortality.

**Figure 4.**
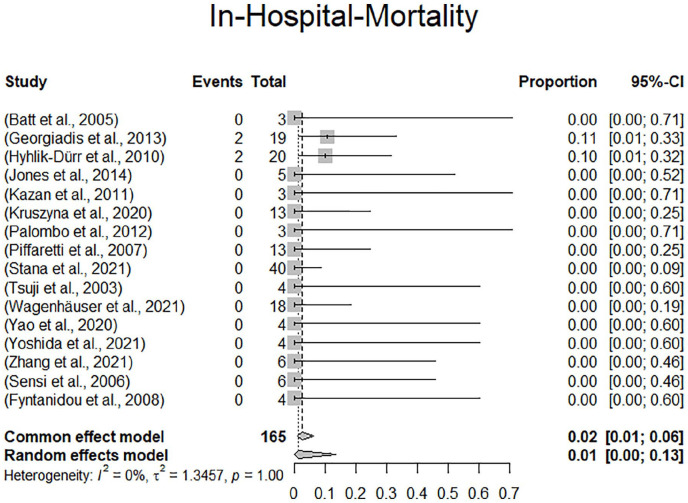
Forest plot displaying in-hospital mortality.

Survival rates at 1 month could be extracted from 13 of 16 studies including 137 of 165 patients (83.0%). Eleven studies reported 100% survival at 1 month (114/137, 83.2%). The remaining studies reported 94.7% (19/137, 13.9%) and 75% (4/137, 2.9%), respectively. Random effects modeling resulted in a pooled mortality at 30 days of 1.0% (confidence interval [CI]: 0%-6.0%). There were 2 deaths reported within 30 days. The 2 deaths occurred in a rupture case with abdominal pain and shock in a 76-year-old male dialysis patient who died from multiple organ dysfunction syndrome within 30 days. The other patient died on day 5 following the index procedure from cardiac and pulmonary events following rupture with hemodynamic instability. There were 2 additional in-hospital deaths reported beyond 1 month following the index procedure, which totals the number of in-hospital deaths to 4 (4/165, 0.6%). Two of the 4 perioperative in-hospital deaths occurred in ruptured patients, the other 2 cases were not specified with regard to initial clinical presentation. In-hospital mortality according to the random effects model was 1.0% (CI: 0.0%-13.0%). The forest plots for 30-day mortality and in-hospital mortality are presented in Figures 3 and 4.

#### Survival at 12 and 36 months

Seven studies reported 12-month survival rates comprising 74/165 patients (44.8%). In 3 studies including 57/74 patients (77.0%) the 12-month survival was reported to range between 77.1% and 89.0%. The remaining 4 studies (17/74, 23.0%) reported 100% survival at 12 months. Five studies reported 36-month survival rates comprising 64/165 patients (38.8%). Two studies including 39/64 patients (60.9%) reported a 67.9% and 69.0% survival rate, 1 study with 18/64 patients (28.1%) reported 89%, and the remaining 2 studies with 7/64 patients (10.9%) reported 100% survival at 36 months. Survival data analysis beyond 36 months as well as random effects modeling of 12-, and 36-month survival data were not performed due to heterogeneity and incompleteness of reporting.

#### Reinterventions

**Figure 5.**
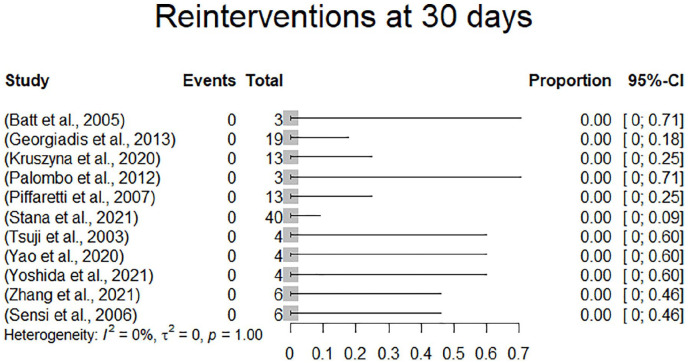
Forest plot displaying reinterventions at 30 days.

Reinterventions were reported in all studies. Among the 165 patients, there were 5 reported reinterventions in 5 patients (3.3%). There were no reported reinterventions within 30 days of follow-up. One patient underwent femoro-femoral bypass surgery for limb occlusion 2 months following the index procedure. There were 3 reinterventions for endoleak. One secondary type 1a endoleak in a severely comorbid patient who was deemed technically unsuited for proximal stent graft extension was treated by embolization of the endoleak as a bailout procedure after 1 year of follow-up, 1 type 1b endoleak was treated by endolining an endovascular aneurysm repair (EVAR) tube graft with an Ovation stent graft during follow-up (TriVascular, Santa Rosa, CA, USA) and one type 3 endoleak was treated by open surgical conversion at 11-month follow-up. One other reintervention was performed for dislocation of 2 stent graft components by balloon dilatation 45 days following the index procedure. In 115 patients, the status with regard to reinterventions at 1 month was extractable. All these studies demonstrated a 0% reintervention rate at 1 month of follow-up. Reinterventions are presented in Table 2. Due to a 0% event rate, there was no effect estimation performed. A forest plot displaying reinterventions at 30 days is presented in Figure 5.

#### Endoleaks

**Figure 6.**
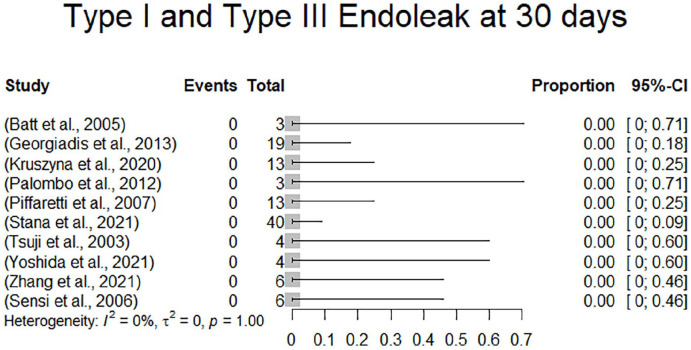
Forest plot displaying endoleaks types 1and 3 at 30 days.

All studies reported on endoleaks. Type 1 and 3 endoleaks undergoing reinterventions were already described above. There was 1 additional type 1a endoleak in 1 of 4 patients receiving the parallel stent graft technique reported by Yao et al. which resolved spontaneously and was no longer detectable at the 3-month follow-up. In summary, there were overall 2 type 1a (1.2%), 1 type 1b (0.6%) and 1 type 3 (0.6%) endoleak, all of which either underwent reintervention or resolved spontaneously during follow-up. In 111 patients, the status with regards to type 1 and type 3 endoleaks at 1-month follow-up was extractable. All these studies exhibited an endoleak type 1 and type 3 rate at 1 month of 0%. Additionally, there were 10 type 2 endoleaks (6.3%). Four of the 10 type 2 endoleaks resolved spontaneously and 1 type 2 endoleak was associated with aortic diameter expansion during follow-up. In 1 patient with type 2 endoleak, the maximum aortic diameter was decreasing during follow-up. The remaining 4 type 2 endoleaks were not described with regards to persistency and/or AAA expansion. There have been no reinterventions reported for type 2 endoleak. Endoleaks are included in Table 4. Due to a 0% event rate, there was no effect estimation performed. A forest plot displaying endoleaks type I and type III is presented in Figure 6. Clinical results are summarized in Table 5.

**Table 5.** Sample Size, Technical Success, Follow-Up, Survival, Overall Mortality, Reinterventions and Endoleaks (Type I and III).

				Follow-up (months)		Overall survival			Mortality		Reinterventions		Endoleaks (I & III)	
ID	Citation	N	Technical success (N, %)	Minimum	Median* or mean^†^	Aa 1 month (%)	At 12 months (%)	At 36 months (%)	Overall (N, %)	In-hospital (N, %)	At 1 month (%)	Overall (N, %)	At 1 month (%)	Overall (N, %)
1	Batt et al.^16^	3	3 (100)	12	26*	100	100	100	0	0	0	0	0	0
2	Georgiadis et al.^17^	19	19 (100)	6	33*	95	86	68	5 (26)	2 (11)	0	2 (11)	0	1 (5)
3	Hyhlik-Dürr et al.^18^	20	20 (100)	0.4	22*	NR	77	69	6 (30)	2 (10)	NR	2 (10)	NR	2 (10)
4	Jones et al.^19^	5	5 (100)	0.03	NR	NR	NR	NR	0	0	NR	0	NR	0
5	Kazan et al.^20^	3	3 (100)	0.5	1*	NR	NR	NR	0	0	NR	0	NR	0
6	Kruszyna et al.^21^	13	13 (100)	6	NR	100	NR	NR	0	0	0	0	0	0
7	Palombo et al.^22^	3	NR	1	NR	100	NR	NR	0	0	0	0	0	0
8	Piffaretti et al.^23^	13	13 (100)	3	24*	100	NR	NR	1 (8)	0	0	0	0	0
9	Stana et al.^24^	40	39 (97.5)	2	14*	100	NR	NR	1 (3)	0	0	0	0	0
10	Tsuji et al.^25^	4	4 (100)	4	14^†^	100	NR	NR	1 (25)	0	0	0	0	0
11	Wagenhäuser et al.^26^	18	18 (100)	NR	19^†^	100	89	89	1 (6)	0	NR	0	NR	0
12	Yao et al.^27^	4	4 (100)	5	NR	100	100	100	0	0	0	0	NR	1 (25)
13	Yoshida et al.^28^	4	4 (100)	12	NR	100	100	NR	0	0	0	0	0	0
14	Zhang et al.^29^	6	NR	3	NR	100	NR	NR	0	0	0	0	0	0
15	Sensi et al.^30^	6	6 (100)	23	NR	100	100	NR	0	0	0	1(17)	0	0
16	Fyntanidou et al.^31^	4	4 (100)	NR	24^†^	75	NR	NR	1 (25)	0	NR	0	NR	0

Abbreviation: NR, Not reported.

Data are presented as N (%). *Median, †Mean.

## Discussion

The present review has demonstrated that the technical success rate of endovascular abdominal PAU repair was estimated high with a pooled technical success rate of 99.0% (CI: 96.0%%-100%) with no reinterventions at 1 month and no type 1 endoleak at 1-month follow-up. These findings are reflective of the convenient applicability of endovascular techniques for abdominal PAU disease, which is a localized aortic pathology. Furthermore, it was demonstrated, that endovascular repair of abdominal PAU is associated with a low-estimated perioperative mortality of 1.0% (CI: 0.0%-13.0%), which is comparable to contemporary results in AAA cohorts and in contrast to previously formulated hypotheses that abdominal PAU patients are susceptible to an increased risk for cardiovascular events due to their overall atherosclerotic burden.^18^ It was also demonstrated that for this entity mid-, and long-term durability as well as clinical data are still lacking over 30 years following the introduction of endovascular aortic repair.^32,33^

While authors generally applied very similar definitions for PAU, most reports lacked a detailed description of underlying morphology including width, depth, and maximum aortic diameter measurements. It is noteworthy, that with the widespread use of computed-tomography angiography, the definition of PAU has morphed from a histopathological definition introduced originally by Stanson et al.^1^ in 1986 to a cross-sectional imaging-derived definition. Histopathologically, PAU is defined by (1) atherosclerosis, (2) penetration of the internal elastic lamina and subsequent formation of (3) intramural hematoma. Based on a literature synopsis in the present review, the key-items of the definition of abdominal PAU that is intuitively applied by most clinicians based on cross-sectional imaging seem to be (1) a craterlike, focal, localized outpouching of contrast material (described “ulcer-like lesion”)^34^ in the (2) presence of calcification, plaque, or calcified plaque with (3) varying extent of intramural hematoma. Additionally, PAU is accompanied by (4) an outpouching of the outer vessel contour in contrast to irregular intraluminal thrombus, which does not protrude beyond the outer contour of the aorta.^35^ This cross-sectional imaging-driven definition can be accounted for in observational studies by reporting of distinct morphological measurements, namely width, depth, and maximum aortic diameter, as has been previously defined.^4^ Within this context, reports should adhere to existing reporting standards. Detailed morphological descriptions are warranted to reduce the risk of selection bias due to imprecise definition to facilitate data aggregation in the future.^8^

Interestingly, approximately one-fifth of patients with PAU were females, which has been described in previous studies to be even up to one-third of patients and supports the distinct conceptual differentiation of PAU from atherosclerotic true AAA disease as a separate clinical entity with a different natural history and overall prognosis.^4^ Therefore, as long as further evidence and specific PAU guidelines are lacking, results from AAA studies should be extrapolated only carefully for abdominal PAU disease. The detailed pathophysiological mechanisms responsible for PAU are not yet conclusively understood. Similar like degenerative AAA disease, PAU is known to be associated with cardiovascular risk factors. As was expected, although reported heterogeneously, cardiovascular risk factors, and atherosclerotic co-manifestations were common findings in the cohort of this review.

With regard to clinical presentation of patients with abdominal PAU, about 50% of patients included in this review presented with symptoms. The most common symptom was abdominal or back pain without signs of rupture. Conclusions with regard to the prognosis of incidental asymptomatic abdominal PAU based on these reports are invalid, since the cohort of this review represents a highly selected group of patients who were referred to vascular centers and underwent endovascular repair. In this highly selected cohort of patients, only about 1 in 10 patients presented with aortic rupture. A recently published study on the prognosis of asymptomatic PAU disease reported an overall benign natural course by demonstrating that asymptomatic PAU is associated with minimal growth over time (<1 mm/year) and that the cumulative incidence of PAU-related complications is estimated at 6.5% at 10 years after diagnosis.^4^

Due to the limited data on the long-term durability of repair as well as the recently reported benign history of asymptomatic PAU disease, recommendations with regard to treatment indications should be made cautiously. The ESVS recommends invasive treatment only in “complicated PAU” as defined above.^3^ Most authors would agree that patients with co-existing extra-aortic hematoma, embolization symptoms, recurrent pain, computed tomography (CT)-angiographic progression, or total diameter increase over time are reasonable candidates for endovascular repair to prevent rupture.^3^ If patients are technically unsuitable for endovascular repair, open repair remains an option. However, abdominal pain requires sufficient differential diagnosis, and the co-presence of abdominal pain and PAU should not prompt premature invasive treatment. Additionally, there is no conclusive evidence on a maximum aortic diameter threshold or an expansion rate in asymptomatic patients to indicate invasive repair. Based on recent insights in the prognosis of asymptomatic PAU and the lack of mid- and long-term outcome data that was illustrated in this review, there is little evidence that supports elective treatment of asymptomatic abdominal PAU based on angio-morphologic characteristics like >20 mm in width or >10 mm in depth early after initial diagnosis. These risk factors for progressive disease were the results of examining thoracic PAU disease and results are not necessarily applicable to abdominal PAU.^36^ Nonetheless, recent evidence also suggests early reimaging within 3 months after initial diagnosis of asymptomatic PAU and annual imaging in the years thereafter if minimal to no growth is detected. In patients with initial ulcer width >20 mm, thrombosed PAU or associated saccular aneurysm, hypertension, hyperlipidemia or diabetes more frequent imaging surveillance seems justified.^4^ However, until higher level evidence is available, the indication to repair an asymptomatic abdominal PAU remains a case-by-case evaluation in a shared decision-making process.

If the indication to repair is made, there are different technical approaches available. Most patients were treated by standard EVAR but more recently the implantation of balloon-expandable covered stents (BeGraft, Bentley InnoMed GmbH, Hechingen, Germany) has also been introduced.^21,24^ The approach being chosen was mostly guided by anatomical assessment and surgeons’ preference and generally considers landing zones, vascular access, PAU location relative to the aortic bifurcation as well as the presence and severity of iliac occlusive disease.^24^ The technique of balloon-expandable covered stents offers the advantages of a lower profile design without unnecessarily treating extensive lengths of aorta. Independent of the methods used, the incidence of endoleak type 1 was low across all studies since there are usually adequate landing zones in focal aortic disease with sufficient sealing. Reintervention rates were also low. Interestingly, the rate of type 2 endoleak was low as well with only 6.3%. There were also no reinterventions for type 2 endoleak reported and only one type 2 endoleak was associated with AAA expansion during follow-up. This might be explained due to the localized character of PAU disease that potentially involves less lumbar arteries than AAAs. As a consequence, there is a lower risk for crossflow endoleaks type 2b, which are associated with an increased risk of type 2 endoleak persistency which is associated with AAA expansion and late adverse events during follow-up.^37^ Severe aorto-iliac atherosclerosis with thrombotic lumbar artery occlusion might also contribute to the finding that in PAU patients compared to AAA patients endoleak type 2 appears to be a less frequent finding. Future studies reporting on outcomes of endovascular repair of infrarenal PAU should include a more detailed description of aortic branch anatomy.

Several limitations of this study need to be considered. While all authors used similar definitions for abdominal PAU, most reports lacked a detailed morphological assessment including with, depth and maximum aortic diameter. As a result, there is potential selection bias due to application of different diagnostic criteria. Additionally, studies from this review originate in general from specialized facilities over a period of 23 years, so that publication bias needs to be considered and generalizations might be invalid. Furthermore, risk factors, comorbidities, technical details as well as clinical outcomes were reported heterogeneously, which naturally limited the quality of data aggregation. Additionally, quality assessment according to the adapted Modified Coleman Methodology Score indicated a low overall quality of existing studies, primarily limited due to small sample sizes, short periods of follow-up and retrospective study designs. Additionally, there are several relevant statistical limitations to this analysis. Since most of the studies included exhibited no events at all or 100% event rates in other outcomes of interest, accurate estimation of pooled treatment effects was not reliably possible. Furthermore, heterogeneity could not be properly quantified in this setting and the produced statistics regarding heterogeneity do not allow for definitive interpretation. All reported pooled effects and forest plots should therefore be treated as illustrative overviews for the outcomes in question and interpreted with care.

## Conclusion

There is low-level evidence on outcomes of endovascular abdominal PAU repair. While in the short-term endovascular repair of abdominal PAU seems safe and effective, mid-term and long-term data are lacking. In the context of a benign prognosis of asymptomatic PAU and lacking standardization in current reporting, recommendations with regard to treatment indications and techniques in asymptomatic PAUs should be made cautiously.

## Supplemental Material

sj-docx-1-jet-10.1177_15266028231157636 – Supplemental material for Systematic Review on Abdominal Penetrating Atherosclerotic Aortic Ulcers: Outcomes of Endovascular Repair Supplemental material, sj-docx-1-jet-10.1177_15266028231157636 for Systematic Review on Abdominal Penetrating Atherosclerotic Aortic Ulcers: Outcomes of Endovascular Repair by Johannes Hatzl, Dittmar Böckler, Jonathan Fiering, Samuel Zimmermann, Moritz Sebastian Bischoff, Eva Kalkum, Rosa Klotz and Christian Uhl in Journal of Endovascular Therapy

sj-docx-2-jet-10.1177_15266028231157636 – Supplemental material for Systematic Review on Abdominal Penetrating Atherosclerotic Aortic Ulcers: Outcomes of Endovascular Repair Supplemental material, sj-docx-2-jet-10.1177_15266028231157636 for Systematic Review on Abdominal Penetrating Atherosclerotic Aortic Ulcers: Outcomes of Endovascular Repair by Johannes Hatzl, Dittmar Böckler, Jonathan Fiering, Samuel Zimmermann, Moritz Sebastian Bischoff, Eva Kalkum, Rosa Klotz and Christian Uhl in Journal of Endovascular Therapy

sj-docx-3-jet-10.1177_15266028231157636 – Supplemental material for Systematic Review on Abdominal Penetrating Atherosclerotic Aortic Ulcers: Outcomes of Endovascular Repair Supplemental material, sj-docx-3-jet-10.1177_15266028231157636 for Systematic Review on Abdominal Penetrating Atherosclerotic Aortic Ulcers: Outcomes of Endovascular Repair by Johannes Hatzl, Dittmar Böckler, Jonathan Fiering, Samuel Zimmermann, Moritz Sebastian Bischoff, Eva Kalkum, Rosa Klotz and Christian Uhl in Journal of Endovascular Therapy
