# Supplementary material for: Systematic Review on Abdominal Penetrating Atherosclerotic Aortic Ulcers: Outcomes of Endovascular Repair
Source: J Endovasc Ther. 2023 Mar 4;31(6):1027–40. doi: 10.1177/15266028231157636 (PMC11552196; doi:10.1177/15266028231157636)
Supplement: sj-docx-1-jet-10.1177_15266028231157636 – Supplemental material for Systematic Review on Abdominal Penetrating Atherosclerotic Aortic Ulcers: Outcomes of Endovascular Repair [file sj-docx-1-jet-10.1177_15266028231157636.docx]

**Pubmed: 1679 hits (24.02.22)**

PAU[tiab] OR PAUs[tiab] OR (penetrat*[tiab] AND (aorta[tiab] OR aortic[tiab] OR atherosclerot*[tiab] OR atheromatous[tiab]) AND ulcer*[tiab]) OR (penetrat*[tiab] AND ulcer*[tiab] AND (abdominal[tiab] OR infrarenal[tiab])) OR (abdominal[tiab] AND (aorta[tiab] OR aortic[tiab]) AND (lesions[tiab] OR pathologies[tiab])) OR ("Aortic Aneurysm, Abdominal"[Mesh] AND "Ulcer"[Mesh])

AND

**(endovascul*[tiab] AND (treatment*[tiab] OR therap*[tiab] OR approach*[tiab] OR stent*[tiab] OR graft*[tiab] OR repair*[tiab] OR surger*[tiab] OR surgical*[tiab] OR procedure*[tiab] OR reconstruct*[tiab] OR technique[tiab])) OR surgery[tiab] OR endostent*[tiab] OR endograft*[tiab] OR endoprosth*[tiab] OR TEVAR[tiab] OR EVAR[tiab] OR "Endovascular Procedures"[Mesh] OR "Vascular Surgical Procedures"[Mesh] OR "Stents"[Mesh] OR "Blood Vessel Prosthesis" [Mesh] OR "Blood Vessel Prosthesis Implantation"[Mesh]**

**NOT (animals [mh] NOT humans [mh])**

**WoS: 881 hits (24.02.22)**

TS = (PAU OR PAUs)

OR TS= (penetrat* NEAR (aorta OR aortic OR atherosclerot* OR atheromatous) NEAR ulcer*)

OR TS= (penetrat* NEAR ulcer* NEAR (abdominal OR infrarenal))

OR TS = (abdominal NEAR (aorta OR aortic) NEAR (lesions OR pathologies))

AND

**TS = (endovascul* NEAR (treatment* OR therap* OR approach* OR stent* OR graft* OR repair* OR surger* OR surgical* OR procedure* OR reconstruct* OR technique))**

**OR TS = (surgery OR endostent* OR endograft* OR endoprosth* OR TEVAR OR EVAR OR stent*)**

**NOT TI = (mice OR mouse OR rats OR animal)**

**Central: 26 hits (24.02.22)**

(PAU OR PAUs):ti,ab,kw

OR (penetrat* AND (aorta OR aortic OR atherosclerot* OR atheromatous) AND ulcer*):ti,ab,kw

OR (penetrat* AND ulcer* AND (abdominal OR infrarenal)):ti,ab,kw

OR = (abdominal NEAR (aorta OR aortic) NEAR (lesions OR pathologies)):ti,ab,kw

OR ("Aortic Aneurysm, Abdominal"[Mesh] AND "Ulcer"[Mesh])

AND

**(endovascul* AND (treatment* OR therap* OR approach* OR stent* OR graft* OR repair* OR surger* OR surgical* OR procedure* OR reconstruct* OR technique))**:ti,ab,kw

**OR (surgery OR endostent* OR endograft* OR endoprosth* OR TEVAR OR EVAR):**ti,ab,kw

**OR "Endovascular Procedures"[Mesh] OR "Vascular Surgical Procedures"[Mesh] OR "Stents"[Mesh] OR "Blood Vessel Prosthesis" [Mesh]**

**EndNote overall: 2586 hits (24.02.22)**

**EndNote Duplicates removed: 1933 hits**
