# Supplementary material for: Systematic Review on Abdominal Penetrating Atherosclerotic Aortic Ulcers: Outcomes of Endovascular Repair
Source: J Endovasc Ther. 2023 Mar 4;31(6):1027–40. doi: 10.1177/15266028231157636 (PMC11552196; doi:10.1177/15266028231157636)
Supplement: sj-docx-2-jet-10.1177_15266028231157636 – Supplemental material for Systematic Review on Abdominal Penetrating Atherosclerotic Aortic Ulcers: Outcomes of Endovascular Repair [file sj-docx-2-jet-10.1177_15266028231157636.docx]

Scoring-System (adapted Version of the Modified Coleman Methodology Score)

Part A: Only one score to be given for each of 7 sections

| 1. Study size: number of patients | | |
| --- | --- | --- |
|  | <20 | 0 |
|  | 20-40 | 4 |
|  | 41-60 | 7 |
|  | >60 | 10 |
| 2. Mean follow up, months | | |
|  | <12 | 0 |
|  | 12-24 | 5 |
|  | 24-36 | 10 |
|  | 36-48 | 15 |
|  | >48 | 20 |
| 3. Surgical approach | | |
|  | Not stated, unclear, or < 90% of subject undergoing the 1 procedure | 0 |
|  | More than 1 method but >90% of subjects undergoing the 1 procedure | 7 |
|  | Single approach used | 10 |
| 4. Type of study | | |
|  | Retrospective cohort study | 0 |
|  | Prospective cohort study | 15 |
| 5. Description of diagnosis | | |
|  | In <80% | 0 |
|  | In >80% | 3 |
|  | In all | 5 |
| 6. Description of surgical technique | | |
|  | Inadequate (not stated, unclear) | 0 |
|  | Fair (technique only stated) | 3 |
|  | Adequate (technique stated, details of surgical procedure given) | 5 |
| Total | | /65 |

Part B: Scores may be given for each option in each the 3 sections if applicable

| 1. Outcome criteria | | |
| --- | --- | --- |
|  | Outcome measures clearly defined | 4 |
|  | Reported either interrater or intrarater reliability | 3 |
|  | Use of outcome criteria that has reported reliability | 3 |
| 2. Description of subject selection process | | |
|  | Selection criteria reported and unbiased | 5 |
|  | Recruitment rate reported <80% | 5 |
|  | Recruitment rate reported >80% | 3 |
|  | Eligible subjects not included in the study satisfactorily accounted for, or 100% recruitment | 5 |
| Total | | /20 |

Total of Part A and Part B =
