# Supplementary material for: Systematic Review on Abdominal Penetrating Atherosclerotic Aortic Ulcers: Outcomes of Endovascular Repair
Source: J Endovasc Ther. 2023 Mar 4;31(6):1027–40. doi: 10.1177/15266028231157636 (PMC11552196; doi:10.1177/15266028231157636)
Supplement: sj-docx-3-jet-10.1177_15266028231157636 – Supplemental material for Systematic Review on Abdominal Penetrating Atherosclerotic Aortic Ulcers: Outcomes of Endovascular Repair [file sj-docx-3-jet-10.1177_15266028231157636.docx]

Supplementary table 1: Devices and configurations

| ID | Citation | N | Devices and configurations |
| --- | --- | --- | --- |
| 1 | Batt et al., 2005^1^ | 3 | EVAR (3)  Aorto-biiliac (2)  Talent (Medtronic, Vascular Inc, Santa Rosa, CA, USA) (1)  Zenith (Cook Medical, Bloomington, IN, USA) (1)  Aorto-uniiliac (1)  Zenith (Cook Medical, Bloomington, IN, USA) (1) |
| 2 | Georgiadis et al., 2013^2^ | 19 | EVAR (12), Other (7)  Aorto-biiliac (9)  Excluder (W.L. Gore & Associates, Inc., Flagstaff, AZ, USA) (4)  Talent (Medtronic, Santa Rosa, CA, USA) (1)  Endurant (Medtronic, Santa Rosa, CA, USA) (4)  Aorto-uniiliac (3)  Endofit (Endomed, Phoenix, AZ, USA) (2)  Talent (Medtronic Vascular Inc, Santa Rosa, CA, USA) (1) |
| 3 | Hyhlik-Dürr et al., 20103 | 20 | EVAR (20)  aorto-biiliac (14) or aorto-uniiliac (6)  Talent, AneuRx or Valiant (Medtronic Vascular Inc., Santa Rosa, CA, USA) (16)  Excluder (W.L. Gore & Associates, Inc., Flagstaff, AZ, USA) (2)  Zenith (Cook Medical, Bloomington, IN, USA) (1)  Lifepath (Edwards Lifesciences, Irvine, CA, USA) (1) |
| 4 | Jones et al., 2014^4^ | 5 | EVAR (4), Other (1)  Aorto-uniiliac (4)  Zenith Renu (Cook Medical, Bloomington, IN, USA) (4) |
| 5 | Kazan et al, 2011^5^ | 3 | EVAR (3)  Aorto-biiliac (3)  AneuRx (Medtronic Vascular Inc., Santa Rosa, CA, USA) (1)  Zenith (Cook Medical, Bloomington, IN, USA) (1)  AneuRx and Talent (Medtronic Vascular Inc., Santa Rosa, CA, USA) (1) |
| 6 | Kruszyna et al., 2020^6^ | 13 | Covered stent (13)  CERAB (BeGraft, Bentley InnoMed, Hechingen, Germany) (9)  Tube (BeGraft, Bentley InnoMed, Hechingen, Germany) (4) |
| 7 | Palombo et al., 2012^7^ | 3 | EVAR (3)  Aorto-biiliac (3)  Zenith (Cook Medical, Bloomington, IN, USA) (2)  Excluder (W.L. Gore & Associates, Inc., Flagstaff, AZ, USA) (1) |
| 8 | Piffaretti et al., 2007^8^ | 13 | NR |
| 9 | Stana et al., 2021^9^ | 40 | Covered stent (40)  CERAB (BeGraft, Bentley InnoMed, Hechingen, Germany) (5)  Tube (BeGraft, Bentley InnoMed, Hechingen, Germany) (35) |
| 10 | Tsuji et al., 2003^10^ | 4 | EVAR (4)  Aorto-aortic (tube) (4)  Gianturco Z-stent (GZV, Cook Inc, Bjaeverskov, Denmark) (4) |
| 11 | Wagenhäuser et al., 2021^11^ | 18 | EVAR (18)  Aorto-biiliac (18)  AFX (Endologix, Irvine, CA, USA) |
| 12 | Yao et al., 2020^12^ | 4 | Parallel covered stents technique (Excluder) (4)  Excluder (W.L. Gore & Associates, Inc., Flagstaff, AZ, USA) (4) |
| 13 | Yoshida et al., 2021^13^ | 4 | EVAR (4)  Aorto-biiliac (4)  AFX (Endologix, inc., Irvine, CA, USA) (4) |
| 14 | Zhang et al., 2021^14^ | 6 | NR |
| 15 | Sensi et al., 2006^15^ | 6 | EVAR (6)  Aorto-aortic (tube) (1)  Zenith (Cook Medical, Bloomington, IN, USA) (1)  Aorto-biiliac (5)  Excluder (W.L. Gore & Associates, Inc., Flagstaff, AZ, USA) (4)  Zenith (Cook Medical, Bloomington, IN, USA) (1) |
| 16 | Fyntanidou et al., 2008^16^ | 4 | EVAR (4)  Aorto-aortic (tube) (1)  Excluder (W.L. Gore & Associates, Inc.; Flagstaff, AZ, USA) (1)  Aorto-biiliac (1)  Excluder (W.L. Gore & Associates, Inc.; Flagstaff, AZ, USA) (1)  Aorto-uniiliac (2)  Endofit (Endomed, Phoenix, AZ, USA) (2) |

NR: Not reported
